# Has the HCV cascade of care changed among people who inject drugs in England since the introduction of direct-acting antivirals?

**DOI:** 10.1016/j.drugpo.2024.104324

**Published:** 2024-01-12

**Authors:** H.D. Gliddon, Z. Ward, E. Heinsbroek, S. Croxford, C. Edmundson, V.D. Hope, R. Simmons, H. Mitchell, M. Hickman, P. Vickerman, J. Stone

**Affiliations:** a Population Health Sciences, Bristol Medical School, University of Bristol, Bristol, United Kingdom; b National Public Health Specialty Training Programme, South West, United Kingdom; c NIHR Health Protection Research Unit in Behavioural Science and Evaluation at University of Bristol, United Kingdom; d Blood Safety, Hepatitis, Sexually Transmitted Infections and HIV Service, UK Health Security Agency, London, United Kingdom; e National Public Health Speciality Training Programme, West Midlands, United Kingdom; f Public Health Institute, Liverpool John Moores University, Liverpool, United Kingdom

**Keywords:** Hepatitis C virus, Testing, Direct acting antivirals, Injecting drug use, People who inject drugs, Hepatitis C elimination, Cascade of care

## Abstract

**Background:**

In England, over 80 % of those with hepatitis C virus (HCV) infection have injected drugs. We quantified the HCV cascade of care (CoC) among people who inject drugs (PWID) in England and determined whether this improved after direct-acting antivirals (DAAs) were introduced.

**Methods:**

We analysed data from nine rounds of national annual cross-sectional surveys of PWID recruited from drug services (2011–2019; *N* = 12,320). Study rounds were grouped as: ‘Pre-DAAs’ (2011–2014), ‘Prioritised DAAs’ (2015–2016) and ‘Unrestricted DAAs’ (2017–2019). Participants were anonymously tested for HCV antibodies and RNA and completed a short survey. We assessed the proportion of PWID recently (current/previous year) tested for HCV. For participants ever HCV treatment eligible (past chronic infection with history of treatment or current chronic infection), we assessed the CoC as: HCV testing (ever), received a positive test result, seen a specialist nurse/doctor, and ever treated. We used logistic regression to determine if individuals progressed through the CoC differently depending on time-period, whether time-period was associated with recent testing (all participants) and lifetime HCV treatment (ever eligible participants), and predictors of HCV testing and treatment in the Unrestricted DAAs period.

**Results:**

The proportion of ever HCV treatment eligible PWID reporting lifetime HCV treatment increased from 12.5 % in the Pre-DAAs period to 25.6 % in the Unrestricted DAAs period (aOR:2.40, 95 %CI:1.95–2.96). There were also increases in seeing a specialist nurse/doctor. The largest loss in the CoC was at treatment for all time periods. During the Unrestricted DAAs period, recent (past year) homelessness (vs never, aOR:0.66, 95 % CI:0.45–0.97), duration of injecting (≤3 years vs >3 years; aOR:0.26, 95 %CI:0.12–0.60), never (vs current, aOR:0.31, 95 %CI:0.13–0.75) or previously being prescribed OAT (vs current, aOR:0.67, 95 %CI:0.47-0.95), and never using a NSP (vs past year, aOR:0.27, 95 %CI:0.08–0.89) were negatively associated with lifetime HCV treatment. The proportion of PWID reporting recent HCV testing was higher during Unrestricted DAAs (56 %) compared to Pre-DAAs (48 %; aOR:1.28, 95 %CI:1.06–1.54).

**Conclusion:**

COC stages from seeing a specialist onwards improved after DAAs became widely available. Further improvements in HCV testing are needed to eliminate HCV in England.

## Introduction

In the UK, as in other high-income countries, the majority (>85 %) of hepatitis C virus (HCV) transmission is through injecting drug use (Harris et al, 2019; Trickey et al, 2019) resulting in high levels of HCV among people who inject drugs (PWID) (Degenhardt et al, 2023). In 2021, an estimated 57 % of PWID in England were HCV antibody-positive, similar to previous years, while the proportion with current HCV infection (RNA positive) had fallen to 14.4 %, down from 28.1 % in 2015 (UKHSA, 2023).

For PWID, HCV prevention has traditionally focused on provision of needle and syringe programmes (NSP) and opioid agonist treatment (OAT), which are effective at reducing HCV transmission risk (Platt et al, 2017). However, these interventions alone are insufficient to substantially reduce HCV incidence (Martin et al, 2013; Vickerman et al, 2012; Ward et al, 2018), with the addition of antiviral therapy being fundamental to reducing incidence (Martin et al, 2013). This has led to a new focus on proactively testing and treating PWID (Public Health England, 2019a).

Now that HCV can be effectively treated using direct-acting antivirals (DAAs), WHO has recommended targets for its elimination as a public health threat by 2030 (World Health Organization, 2016a). These targets include an 80 % reduction in incidence, and a reduction in HCV-related mortality of 65 % by 2030, compared to 2015 levels (World Health Organization, 2016b). The UK committed to achieving these targets by 2030 in May 2016, with NHS England committing in 2018 to an accelerated goal of elimination by 2025 (NHS England, 2019).

UK guidelines state that people with chronic HCV infection should be under the care of a hepatologist or specialist gastroenterologist, supported by specialist nurses, and should be considered for antiviral therapy, which is always initiated by a specialist (NICE, 2022). Since 2004, clinical guidelines in the UK have recommended that ongoing injecting drug use is not a contraindication to HCV treatment. In 2015, DAAs became the standard of care treatment in England but were prioritised to patients with compensated cirrhosis or advanced liver disease (NHS England, 2015). Restrictions for treatment based on disease stage were lifted in 2017.

A recent study among PWID in England demonstrated that viremic infection is associated with a history of homelessness or incarceration (Bardsley et al, 2021). Vulnerable and marginalised PWID, including those who are currently or have ever been homeless or incarcerated, are at higher risk of HCV acquisition (Arum et al, 2021; Stone et al, 2018) and may have less contact with health services (Lazarus et al, 2019).

To inform national HCV elimination strategies, it is important to understand which groups are not being treated for HCV. A nationally representative cascade of care was established in the pre-DAA era to assess drop out of HCV-infected patients from the care pathway, between testing/diagnosis and either completing treatment or sustained virological response (Ireland et al, 2019; Simmons et al, 2018). Analysing the current cascade of care is important to identify gaps in the cascade and factors associated with HCV testing and treatment uptake. This study aimed to address these questions for PWID in England, to determine whether the cascade of care has improved since the introduction of DAA treatments, and assess whether more marginalised PWID (i.e. those with a history of incarceration or homelessness and those not engaging in NSP or OAT (Harris et al, 2015)) are less likely to be tested and treated.

## Methods

### Data source and participants

We used data from the Unlinked Anonymous Monitoring (UAM) Survey of PWID. This annual cross-sectional survey is delivered across England, Wales and Northern Ireland through specialist agencies that provide a range of services to individuals who inject psychoactive drugs, from medical treatment to needle and syringe programmes and outreach work. (Cullen et al, 2015). Participants complete a short self-administered behavioural questionnaire and provide a dried blood spot (DBS) sample for HIV, hepatitis B virus and HCV testing (Public Health England, 2019b), which was conducted using previously described methods. Limited demographic data is collected, and responses are anonymous. The services collaborating in the UAM Survey are geographically spread across the 22 Operational Delivery Networks (ODNs) in England (ODNs are regional hubs that co-ordinate HCV treatment delivery in England), as well as in Wales and Northern Ireland.

### Data analysis

Data for participants in England from the 2011 to 2019 rounds of the UAM Survey were used. We restricted our analyses to those who reported injecting drugs in the past year, had not completed the UAM Survey in a previous year, and had complete HCV testing data from their DBS sample. Participants’ HCV antibody and RNA status was assessed through testing of the DBS samples.

Survey years were divided into three time periods: 2011–2014 were defined as ‘Pre-DAAs’, before DAAs were widely available; 2015–2016 as ‘Prioritised DAAs, when DAAs were the standard of care but treatment was still restricted based on disease stage (Harrison et al, 2019), and 2017–2019 as ‘Unrestricted DAAs’, characterised by wider availability of DAAs with no HCV disease stage eligibility criteria (Radley et al, 2019). Additional analyses were conducted using data from 2018-2019 only to include responses to questions regarding recent incarceration (incarceration history categorised as never incarcerated, incarcerated in last year, or previously incarcerated but not in last year).

### Cascade of care

We present the proportion ever or recently (within the current or previous year) tested for HCV for each time period for all PWID (regardless of HCV status) in this study, according to DAA time period.

**Fig. 1:**
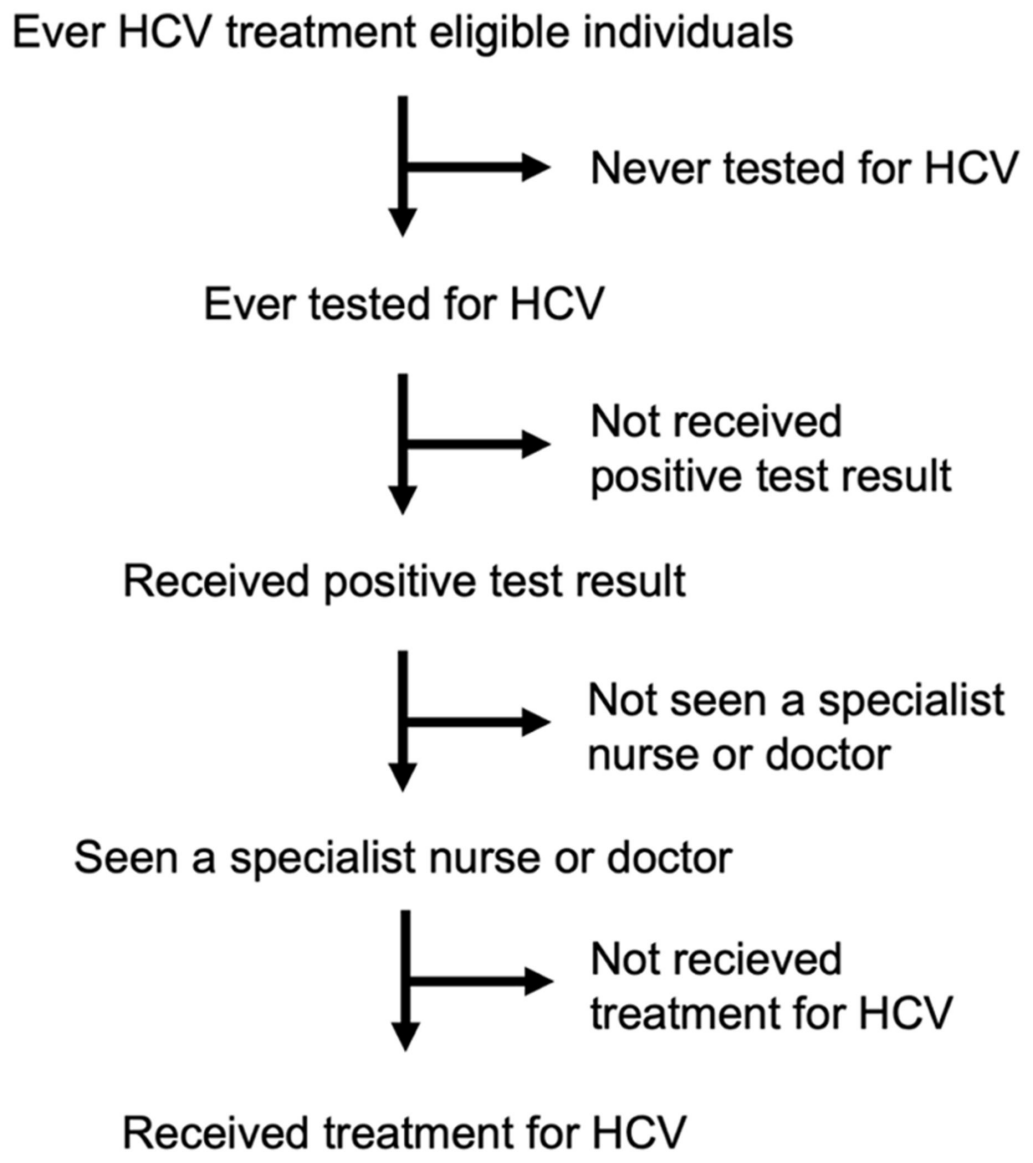
HCV Cascade of care.

For each time period, we described the HCV cascade of care among those ever eligible for HCV treatment; that is those with current active infection (i.e. HCV RNA positive) or those with past infection (HCV antibody positive and RNA negative) who had a history of treatment. The cascade was defined as: ever tested for HCV, ever received a positive HCV diagnosis (2011–16 defined as result of last HCV test was positive; 2017–19 defined as result of last HCV test was antibody positive), ever seen a specialist nurse or doctor, and ever treated for HCV (Fig. 1). This information was self-reported within the questionnaire (Table S1).

### Statistical analysis

Stata Version 17.0 was used for data analysis. Demographic and HCV-related behaviours were tabulated across study periods. We tested for increases in each stage of the cascade of care by DAA time period and investigated the effects of homelessness, NSP use and OAT prescription on the progression of individuals ever eligible for HCV treatment through the cascade of care in the Unrestricted DAAs time period. Using logistic regression, we evaluated whether DAA period was associated with increased HCV treatment and testing uptake after adjusting for individual-level characteristics and clustering by ODN.

For the Unrestricted-DAAs period, we also performed logistic regression analyses to assess whether homelessness (in the past year, previously but not in past year, or never) incarceration history (ever been in prison), NSP use (in the past year, previously but not in past year, or never), and OAT prescription history (currently prescribed, previously prescribed but not currently, or never prescribed) were associated with a history of HCV treatment or recent HCV testing. Based on prior research (Yuan et al, 2022), we also assessed whether recent (last 12 months) use of different healthcare services (Accident and Emergency, GP sexual health, walk-in, pharmacy, dentist and prison-health) were associated with recent HCV testing. Each healthcare service was operationalised as a binary variable. We fitted univariable and multi-variable logistic regression models whilst accounting for clustering by ODN. All variables from the univariable analyses were considered in the multivariable analyses. We adjusted for recent initiation of injecting drugs (in the last three years) as a potential confounder in both analyses and also adjusted for age in analyses for lifetime HCV treatment. To assess the potential for multicollinearity in our models, we computed variance inflation factors (VIFs). In all models, the VIFs did not exceed 4. We used a complete case analysis strategy for missing data.

## Results

### Sample demographics and HCV-related characteristics

A total of 12,320 survey responses from PWID were analysed in this study. Table 1 shows how demographic and HCV-related characteristics differed over the time periods. The distribution of age of respondents advanced with time, with more than double the proportion of respondents in the older age group (50 years and over) in the Unrestricted DAAs years (11.8 %), compared to the Pre-DAAs years (5.0 %). An increase in recent (past year) homelessness was observed in the Unrestricted DAAs years (50.6 %), compared to both the prioritised DAA years (43.1 %) and the Pre-DAAs years (39.8 %). There was also evidence of increasing proportion of PWID currently prescribed OAT over time. The number of participants who were HCV antibody-positive increased with time, likely linked to the ageing (and increasing duration of injecting) of respondents, with 49.2 % in the Pre-DAAs years, 55.0 % in the Prioritised DAAs years, and 58.6 % in the Unrestricted DAAs years. Participants were enrolled within ODNs across England, with the highest proportion from the North East and Cumbria (10.7 %), followed by Birmingham (10.5 %) (Supporting information, Table S2).

### Trends in HCV testing over time

**Fig. 2:**
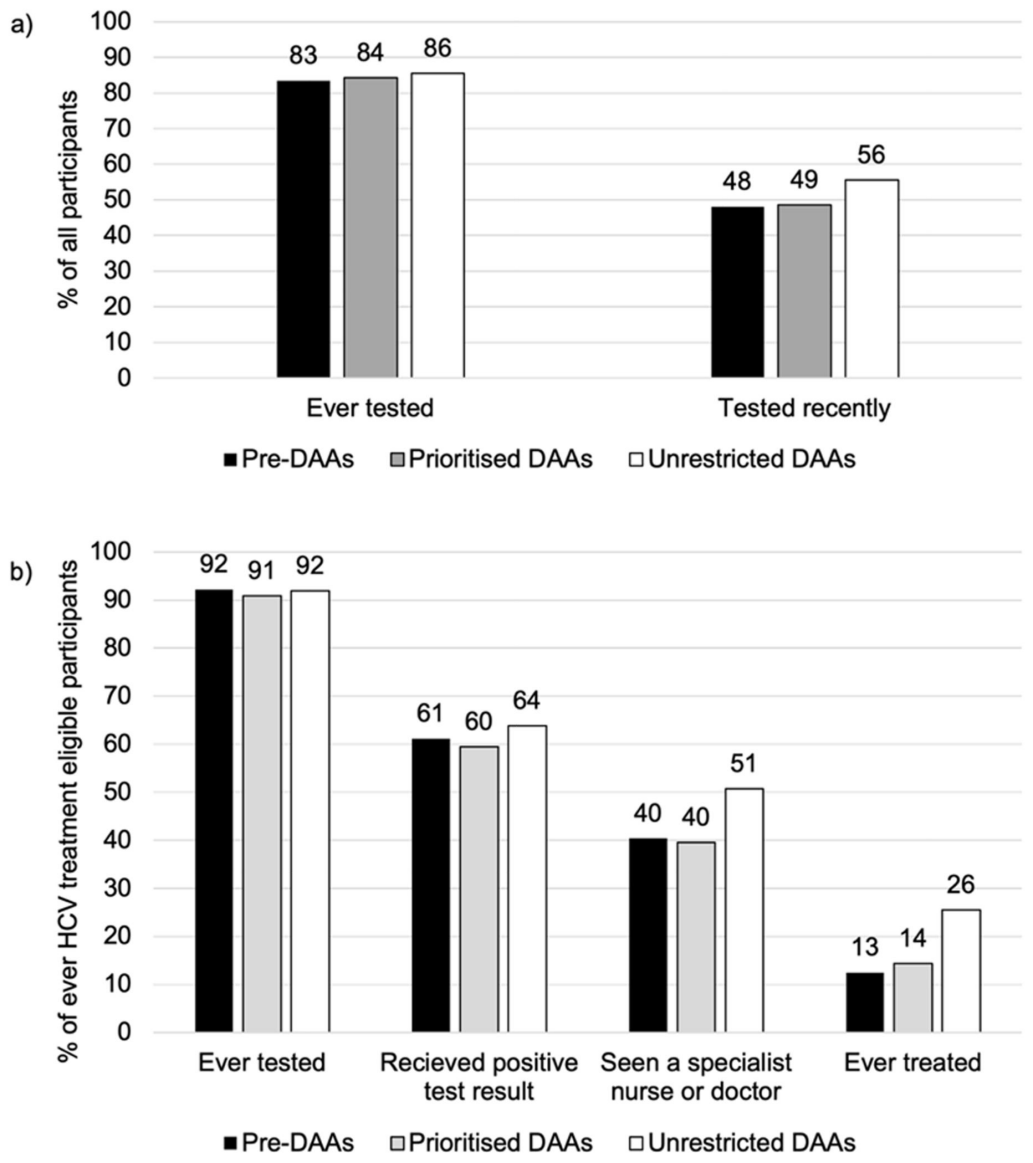
(a) Changes over time in the proportion of all participants reporting ever and recently testing for HCV, by DAA time period. (b) Cascade of care for participants ever HCV treatment eligible, by DAA time period.

The percentage of respondents reporting having ever tested for HCV increased from 84.1 % in 2011 to 87.6 % in 2019. The proportion reporting a recent HCV test (in the current or previous year) remained stable between the Pre-DAAs period and Prioritised DAAs period at 48.1–48.6 % (OR of Prioritised DAA vs Pre-DAA: 1.02, 95 %CI 0.92–1.13), but then increased to 55.6 % (OR 1.35, 95 %CI 1.23–1.48) in the Unrestricted DAAs period (Fig. 2a). After adjusting for individual-level characteristics and ODN, participants in the Unrestricted DAAs period were more likely to have recently been tested for HCV compared to participants in the Pre-DAAs time period (aOR 1.28, 95 %CI 1.06–1.54) (Fig. 2a).

### Predictors of recent HCV testing in the Unrestricted DAAs period

Past (aOR 1.36, 95 %CI 1.07–1.73) or recent (aOR 1.52, 95 %CI 1.27–1.81) homelessness, and past year use of accident and emergency (A&E) (aOR 1.20, 95 %CI 1.02–1.40), sexual health (aOR 1.59, 95 %CI 1.17–2.17), walk-in (aOR 1.16, 95 %CI 1.02–1.33) or prison health (aOR 1.62, 95 %CI 1.36–1.93) services were associated with increased odds of recent testing (Table 2). Conversely, compared to those currently prescribed OAT, PWID who had never been prescribed OAT (aOR 0.51, 95 %CI 0.41–0.64) or had been previously prescribed OAT (aOR 0.67, 95 % CI 0.54–0.84) were less likely to report recent HCV testing. Similarly, those who had never (aOR 0.56, 95 %CI 0.40–0.80) or had previously used an NSP (aOR 0.72, 95 %CI 0.54–0.96) were less likely to report recent HCV testing than PWID who had used an NSP in the past year (Table 2). Using data from 2018/2019, which included responses to additional survey questions, recent HCV testing was higher among those reporting incarceration in the past year (aOR 1.44, 95 %CI 1.13–1.82) compared to those never incarcerated (Table S3).

### Trends in HCV cascade of care over time

Among the participants identified as ever eligible for HCV treatment (*n* = 3227), 9.4 % reported ever receiving treatment in 2011, rising to 23.6 % in 2019. A significantly higher proportion reported that they had seen a specialist nurse or doctor, and ever been treated in the Unrestricted DAAs period compared to the Pre-DAAs period (Table S4 and Fig. 2b). There was no significant difference in any step of the cascade between the Pre-DAAs period and Prioritised DAAs period. The largest drop off in this cascade for all time periods was at treatment, with half (50.4 %) of ever HCV treatment eligible individuals who had seen a specialist nurse or doctor reporting ever being treated in the Unrestricted DAAs period, with this increasing from 31.0 % and 36.4 % in the Pre-DAAs and Prioritised DAAs periods respectively. Although there were small increases in the proportion of participants who had seen a specialist nurse or doctor (from 40.4 % Pre-DAAs to 50.7 % Unrestricted DAAs),the proportion reporting having received treatment doubled from 12.5 % in the Pre-DAAs period, to 14.4 % (OR 1.17, 95 %CI 0.91–1.52) in the Prioritised DAAs period, and then to 25.6 % (OR 2.40, 95 %CI 1.95–2.96) in the Unrestricted DAAs time period. After adjusting for individual-level characteristics and ODN, participants in the Unrestricted DAAs period were more likely to have received HCV treatment compared to participants in the Pre-DAAs period (adjusted odds ratio (aOR) 2.47, 95 %CI 1.72–3.54).

Compared to those who had never been homeless, a significantly higher proportion of those who had previously (not in the past year) been homeless reported ever testing, receiving a positive test result, and seeing a specialist nurse or doctor in the Unrestricted DAAs years (Figure S2a and Table S5). Although those reporting recent homelessness were more likely to report ever testing for HCV than those who had never been homeless, they were less likely to report receiving HCV treatment. Compared to those who were currently or had previously been prescribed OAT, those who had never been prescribed OAT (7.3 % of participants) were less likely to report engagement in each step of the cascade of care (Figure S2b and Table S6). Those who had previously (not currently) been prescribed OAT were less likely to report ever receiving HCV treatment compared to those who were currently prescribed OAT. Compared to those who had used NSP in the last year, those who had never used a NSP were less likely to have ever been tested, whilst those who had previously used a NSP were lore more likely to have ever been treated (Figure S2c and Table S7).

### Predictors of HCV treatment among in the Unrestricted DAAs period

Participants who reported recent homelessness (compared to never; aOR 0.66, 95 %CI 0.45–0.97), never being prescribed OAT (compared to current use; aOR 0.31, 95 %CI 0.13–0.75) or previously being prescribed OAT (compared to current use; aOR 0.67, 95 %CI 0.47–0.95), never using a NSP (compared to past year use; aOR 0.27, 95 % CI 0.08-0.89), and being a recent initiate to injecting (within past 3 years; aOR 0.26, 95 %CI 0.12–0.60) were less likely to report HCV treatment (Table 3). Age and incarceration history were not significantly associated with HCV treatment in the adjusted analysis (Table 3).

## Discussion

Although UK guidelines recommend that HCV testing is provided annually to those that are at risk of infection, we found that only half of PWID had been tested in the current or previous year with only modest improvements (from 48 % to 56 %) over 2011–2019. Despite this, there was clear evidence that HCV treatment uptake has increased more than two-fold since DAAs became widely available (2017–19).

Previous studies have provided useful baselines in the HCV cascade of care prior to the introduction of DAAs, including the effects of age and other factors on evidence of HCV treatment (Simmons et al, 2018) and the impacts of repeat testing and linkage to care on treatment rates (Ireland et al, 2019), but have not assessed whether the cascade of care has changed over recent years. We found that for PWID, seeing a specialist nurse or doctor and receiving treatment have improved since DAAs became available. In all DAA time periods, the largest loss on the care pathway to HCV treatment was at the treatment stage. However, there were substantial improvements over time; with the proportion of people who had seen a specialist nurse or doctor who had ever been treated increasing from 31 % in the Prioritised DAAs period to 50 % in the Unrestricted DAAs period. Further data from the UAM, only available since 2017, shows that a quarter of those who had seen a specialist nurse or doctor had been offered treatment but did not uptake treatment. This could be because participants had been offered interferon based treatment prior to the introduction of DAAs or, among those offered DAAs, that some patients still hold perceptions of treatment based on older interferon based treatments (All-Party Parliamentary Group, 2018).

Several analyses have considered the HCV cascade of care and factors associated with HCV testing and/or treatment among PWID in other settings (Iversen et al, 2020, 2017; Mirzazadeh et al, 2021; Valerio et al, 2020; Young et al, 2018). Using similar study designs to the UAM, repeat surveys of PWID in Australia and Scotland suggest that HCV treatment also increased since the introduction of DAAs in those settings (Iversen et al, 2020; Palmateer et al, 2021). These studies suggest that increases in HCV treatment in England are similar to those in Scotland, where the proportion of PWID ever treated doubled between 2013/14 and 2017/18, but are smaller than those in Australia, where this proportion increased six-fold between 2010 and 2019.

We found that participants who had been homeless in the year preceding survey completion were more likely to have been tested for HCV but less likely to have been treated for HCV. Previous international analyses have also found that HCV treatment uptake among PWID is lower among those who are homeless (Corcorran et al, 2021; Valerio et al, 2020). Homelessness also increases HCV acquisition risk among PWID (Arum et al, 2021) and is estimated to contribute substantially to HCV transmission among PWID in England and other high-income settings (Stone et al, 2022). It is therefore vital that interventions are developed to reduce transmission and improve treatment uptake among this marginalised population if HCV elimination goals are to be achieved. Such interventions are likely to have significant population benefits and be cost-effective (Ward et al, 2019).

NSP and OAT use were strong predictors of HCV testing and treatment uptake in our analysis, suggesting that these services are providing important new pathways for PWID receiving them or are referring PWID to HCV treatment. These findings agree with a recent systematic review which demonstrated that OAT is associated with greater HCV testing and treatment uptake (Grebely et al, 2021). Recent analyses on the 2019 UAM survey for England, Wales and Northern Ireland found that general healthcare use was associated with HCV testing (Yuan et al, 2022). Our study adds to this, showing that since 2017, HCV testing uptake was only higher among those using sexual, A&E, walk-in or prison health services but not other health services (GP, dentist, or a pharmacy). This suggests that new initiatives focussed on providing HCV testing in GPs and pharmacies (NHS England, 2020, 2022), which have been shown to be highly effective models in the UK (Radley et al, 2020; Roberts et al, 2020), may enable increases in the reach of HCV testing and so levels of HCV diagnosis.

Other recent analyses of the UAM Survey have found that the proportion of antibody-positive PWID who have chronic infection decreased in 2017/18 compared to previous years (Bardsley et al, 2021), consistent with our findings that treatment expanded since DAAs became widely available. These analyses also found that a history of homelessness and incarceration were associated with chronic infection. Although this aligns with homeless PWID being less likely to have ever been treated in our study, we found no significant differences by incarceration history. Our findings that a history of incarceration was associated with recent HCV testing but not HCV treatment aligns with previous research that suggested pathways from testing to treatment in prisons are sub-optimal (Bryce et al, 2023; Mohamed et al, 2020). However, our analyses likely do not capture the effects of recent prison test and treat initiatives, that through improving testing at reception (starting from May 2019) (Mongale et al, 2023) or conducting high intensity test and treat events (starting from July 2019) (HCV Action, 2023), have been shown to be highly effective, achieve high rates of treatment initiation, and able to achieve micro-elimination with prisons. Previous international modelling suggests that HCV treatment in prisons is likely to be key for achieving HCV elimination among PWID (Stone et al, 2023) and that mass HCV screening and treatment events can be cost-effective (Ward et al, 2021), but research is required to determine whether these initiatives can contribute to improving the overall cascades of care for PWID and the frequency of mass HCV screening and treatment events needed to maintain HCV micro-elimination within prisons.

### Strengths and limitations

Participation in the UAM Survey relies on individuals engaging with low threshold and other specialist drugs services, so will exclude people who are not in regular contact with these services and the UAM is a comparatively small sample of overall PWID population in England (Larney et al, 2017). Nonetheless recruitment through a diverse range of settings reduces potential bias, with previous research suggesting UAM survey participants are broadly representative of the wider PWID population (Hickman et al, 2007). However, there is sampling variability between years, with each local authority represented on average in 5 study rounds during our study period, which could explain some differences in HCV testing and the cascade of care over the years. Additionally, to preserve anonymity, we did not have data on sex and so could not investigate differences between male and female PWID (approximately a quarter of UAM respondents are female (Trickey et al, 2018)).

The UAM behavioural questionnaire relies on participants’ self-report, which could be subject to recall bias. This bias may have been exacerbated by survey questions for each step of the cascade only be asked if participants had answered yes to completing the previous step. However, given the historically low levels of treatment, we anticipate that this would not have greatly affected our findings, particularly of an increasing trend in the proportions who have received HCV treatment.

We did not include data for the 2020 round of the UAM Survey because sampling and participation differed during the COVID-19 pandemic (Public Health England, 2021). The effects of the COVID-19 pandemic on HCV testing and treatment were wide-ranging and varied, including significant disruption of NSP, OAT and other services for PWID (Croxford et al, 2021; Whitfield et al, 2020). Future studies should evaluate how the pandemic affected the HCV cascade of care among PWID.

## Conclusions

This study shows that while HCV treatment and testing have increased over time, there is no evidence of increasing diagnosis among PWID. The increases in treatment could be limited if they are not accompanied by further improvements in HCV testing and diagnosis rates. To achieve HCV elimination among PWID, HCV testing rates must improve, while the linkage to treatment needs to be improved among PWID who are homeless or not engaged in harm reduction services.

## Supplementary Material

Supplementary materials

## Figures and Tables

**Table 1:** Demographic and HCV-related characteristics of UAM Survey respondents. Data derived from survey responses and DBS testing.

		Pre-DAAs 2011–2014 *N =* 5755	Prioritised DAAs 2015–2016 *N =* 2616	Unrestricted DAAs 2017–2019 *N =* 3949
Age, years	18–29	25.0 %	15.6 %	11.1 %
	30–39	44.2 %	43.1 %	41.8 %
	40–49	25.9 %	32.6 %	30.4 %
	50+	5.0 %	8.7 %	11.8 %
Ever been homeless	No	20.9 %	22.3 %	20.6 %
	Yes, not in past year	39.3 %	34.7 %	28.8 %
	Yes, in past year	39.8 %	43.1 %	50.6 %
Ever been in prison Used an NSP	No	28.6 %	32.3 %	31.3 %
	Yes	71.4 %	67.7 %	68.7 %
	Never	4.6 %	5.5 %	4.7 %
	Yes, not in past year	10.9 %	8.2 %	8.9 %
	Yes, in past year	84.5 %	86.3 %	86.4 %
Ever prescribed OAT	No	17.5 %	16.2 %	7.3 %
	Yes, not currently prescribed	16.2 %	15.4 %	15.7 %
	Yes, currently prescribed	66.3 %	68.5 %	77.0 %
First injected during the preceding three years	No	86.4 %	88.9 %	88.3 %
	Yes	13.6 %	11.1 %	11.7 %
HCV Ab Prevalence (from DBS result)	Positive	49.2 %	55.0 %	58.6 %
	Negative	50.8 %	45.0 %	41.4 %
Used a GP in past year	No	28.0 %	27.0 %	33.3 %
	Yes	72.0 %	73.0 %	66.8 %
Used A&E in past year	No	65.1 %	65.6 %	65.1 %
	Yes	34.9 %	34.4 %	34.9 %
Used a sexual health service in the past year	No	87.6 %	88.8 %	94.0 %
	Yes	12.4 %	11.2 %	6.0 %
Used walk-in service in past year	No	77.5 %	78.6 %	76.2 %
	Yes	22.5 %	21.4 %	23.8 %
Used pharmacy in past year	No	–	–	55.6 %
	Yes	–	–	38.4 %
	Missing		–	6.0 %
Used a dentist in past year	No	–	–	66.1 %
	Yes	–	–	27.0 %
	Missing	–	–	6.8 %
Used prison health service in past year	No	–	–	74.1 %
	Yes	–	–	19.1 %
	Missing	–	–	6.8 %

**Table 2:** Unadjusted and adjusted odds ratios with 95 % confidence intervals for HCV testing among all participants in the Unrestricted DAAs period (*N* = 2791).

		OR (95 % CI)	P- Value	aOR (95 % CI)	P-value
Ever been homeless	No	Ref		Ref	
	Yes, not in past year	1.46 (1.18–1.81)	<0.001	1.36 (1.07–1.73)	0.011
	Yes, in past year	1.65 (1.43–1.89)	<0.001	1.52 (1.27–1.81)	<0.001
Ever been in prison	No	Ref		Ref	
	Yes	1.41 (1.24–1.61)	<0.001	1.02 (0.86–1.21)	0.822
Ever used a needle and syringe programme	Yes, in past year	Ref		Ref	
	Yes, not in past year	0.76 (0.58–1.01)	0.060	0.72 (0.54–0.96)	0.024
	No	0.47 (0.36–0.62)	<0.001	0.56 (0.40–0.80)	0.001
Ever prescribed OAT	Yes, currently prescribed	Ref		Ref	
	Yes, not currently prescribed	0.73 (0.58–0.91)	0.005	0.67 (0.54–0.84)	<0.001
	No	0.45 (0.36–0.56)	<0.001	0.51 (0.41–0.64)	<0.001
First injected during preceding three years	More than three years ago	Ref		Ref	
	In last three years	0.73 (0.59–0.89)	0.002	0.93 (0.72–1.19)	0.561
Used a GP in past year		1.14 (0.97–1.35)	0.117	1.07 (0.88–1.29)	0.506
Used A&E in past year		1.33 (1.16–1.52)	<0.001	1.20 (1.02–1.40)	0.027
Used sexual health service in past year		1.59 (1.20–2.10)	0.001	1.59 (1.17–2.17)	0.003
Used walk-in service in past year		1.24 (1.12–1.39)	<0.001	1.16 (1.02–1.33)	0.027
Used pharmacy in past year		1.22 (1.04–1.42)	0.013	1.09 (0.93–1.27)	0.305
Used a dentist in past year		1.12 (0.97–1.30)	0.123	1.05 (0.93–1.19)	0.424
Used prison health service in past year		1.74 (1.51–2.00)	<0.001	1.62 (1.36–1.93)	<0.001

**Table 3:** Unadjusted and adjusted odds ratios with 95 % confidence intervals for HCV treatment among individuals ever eligible for HCV treatment, in the Unrestricted DAAs period. (*N* = 980).

		OR (95 % CI)	P- Value	aOR (95 % CI)	P-value
Age group	18–29	Ref		Ref	
	30–39	0.87 (0.51–1.46)	0.591	0.81 (0.47–1.37)	0.426
	40–49	1.00 (0.57–1.76)	0.994	0.80 (0.44–1.44)	0.449
	50+	2.10 (1.04–4.26)	0.040	1.67 (0.84–3.31)	0.144
Ever been homeless	No	Ref		Ref	
	Yes, not in past year	1.12 (0.79–1.59)	0.527	1.08 (0.73–1.59)	0.696
	Yes, in past year	0.59 (0.44–0.80)	0.001	0.66 (0.45–0.97)	0.034
Ever been in prison	No	Ref		Ref	
	Yes	1.01 (0.74–1.37)	0.943	0.93 (0.69–1.27)	0.669
Ever used a needle and syringe programme	Yes, in past year	Ref		Ref	
	Yes, not in past year	1.62 (1.06–2.47)	0.025	1.36 (0.89–2.01)	0.158
	No	0.63 (0.26–1.51)	0.297	0.27 (0.08–0.89)	0.032
Ever prescribed OAT	Yes, currently prescribed	Ref		Ref	
	Yes, not currently prescribed	0.59 (0.41–0.85)	0.004	0.67 (0.47–0.95)	0.027
	No	0.24 (0.11–0.52)	<0.001	0.31 (0.13–0.75)	0.009
First injected during preceding three years	More than three years ago	Ref		Ref	
	In last three years	0.21 (0.95)	<0.001	0.26 (0.12–0.60)	0.001
