# Supplementary material for: Has the HCV cascade of care changed among people who inject drugs in England since the introduction of direct-acting antivirals?
Source: Int J Drug Policy. Author manuscript; Available in PMC 2025 Oct 1. (PMC7616700; doi:10.1016/j.drugpo.2024.104324)
Supplement: Supplementary materials [file EMS199261-supplement-Supplementary_materials.docx]

**Table S1:** Questions used to derive HCV cascade of care.

| Cascade of care stage |  | 2011 | 2012 | 2013 | 2014 | 2015 | 2016 | 2017 | 2018 | 2019 |
| --- | --- | --- | --- | --- | --- | --- | --- | --- | --- | --- |
| Ever tested | Eligibility for question | All Participants | All Participants | All Participants | All Participants | All Participants | All Participants | All Participants | All Participants | All Participants |
|  | Question | Have you **ever** had a blood test for hepatitis C? | Have you **ever** had a blood test for hepatitis C? | Have you **ever** had a blood test for hepatitis c? | Have you **ever** had a blood test for hepatitis c? | Have you **ever** had a blood test for hepatitis C? | Have you **ever** had a blood test for hepatitis C? | Have you **ever** had a test for hepatitis C? | Have you **ever** had a test for hepatitis C? | Have you **ever** had a test for hepatitis C? |
|  | Possible Responses | Yes; No | Yes; No | Yes; No | Yes; No | Yes; No | Yes; No | Yes; No | Yes; No | Yes; No |
| Received positive test result | Eligibility for question | Participants who report ever having a test for HCV | Participants who report ever having a test for HCV | Participants who report ever having a test for HCV | Participants who report ever having a test for HCV | Participants who report ever having a test for HCV | Participants who report ever having a test for HCV | Participants who report ever having a test for HCV | Participants who report ever having a test for HCV | Participants who report ever having a test for HCV |
|  | Question | What was the result of your **last** test? | What was the result of your **last** test? | What was the result of your **last** test? | What was the result of your **last** test? | What was the result of your **last** test? | What was the result of your **last** test? | What was the result of your **last** test? | What was the result of your **last** test? | What was the result of your **last** test? |
|  | Possible Responses | Positive;  Negative;  Awaiting result | Positive;  Negative;  Awaiting result | Positive;  Negative;  Awaiting result | Positive;  Negative;  Awaiting result | Positive;  Negative;  Awaiting result | Positive;  Negative;  Awaiting result | Current infection (RNA and antibody **positive**);  Past infection (antibody positive only);  Positive, not sure if current or past infection;  Negative (RNA and antibody negative);  Awaiting result | Current infection (RNA and antibody **positive**);  Past infection (antibody positive only);  Positive, not sure if current or past infection;  Negative (RNA and antibody negative); Awaiting result | Current infection (RNA and antibody **positive**);  Past infection (antibody positive only);  Positive, not sure if current or past infection;  Negative (RNA and antibody negative);  Awaiting result |
| Seen a specialist nurse or doctor | Eligibility for question | Answer to question above was: Positive | Answer to question above was: Positive | Answer to question above was: Positive | Answer to question above was: Positive | Answer to question above was: Positive | Answer to question above was: Positive | Answer to question above was: Current infection/past infection/positive infection and not sure if current or past | Answer to question above was: Current infection/past infection/positive infection and not sure if current or past | Answer to question above was: Current infection/past infection/positive infection and not sure if current or past |
|  | Question | Have you **ever** seen a specialist nurse or doctor (e.g. a hepatologist) about your hepatitis C? | Have you **ever** seen a specialist nurse or doctor (e.g. a hepatologist) about your hepatitis C? | *If tested positive*, have you **ever** seen a specialist nurse or doctor (e.g. a hepatologist) about your hepatitis C? | *If tested positive*, have you **ever** seen a specialist nurse or doctor (e.g. a hepatologist) about your hepatitis C? | *If tested positive*, have you **ever** seen a specialist nurse or doctor (e.g. a hepatologist) about your hepatitis C? | *If tested positive*, have you **ever** seen a specialist nurse or doctor (e.g. a hepatologist) about your hepatitis C? | *If current or past infection, or not sure,* have you **ever** seen a hepatitis nurse or hepatitis doctor (e.g. a hepatologist) about your hepatitis C? | *If current or past infection, or not sure,* have you **ever** seen a hepatitis nurse or hepatitis doctor (e.g. a hepatologist) about your hepatitis C? | *If current or past infection, or not sure,* have you **ever** seen a hepatitis nurse or hepatitis doctor (e.g. a hepatologist) about your hepatitis C? |
|  | Possible Responses | No; Yes, and been **given** medicine for hepatitis C; Yes, but **not** given any medicine for hepatitis C | No; Yes, and been **given** medicine for hepatitis C; Yes, but **not** given any medicine for hepatitis C | No; Yes, and been **given** medicine for hepatitis C; Yes, but **not** given any medicine for hepatitis C | No; Yes, and been **given** medicine for hepatitis C; Yes, but **not** given any medicine for hepatitis C | No; Yes, and been **given** medicine for hepatitis C; Yes, but **not** given any medicine for hepatitis C | No; Yes, and been **given** medicine for hepatitis C; Yes, but **not** given any medicine for hepatitis C | No; Yes, but not offered hepatitis C treatment;  Yes, offered hepatitis C treatment but didn’t take treatment; Yes, offered hepatitis C treatment and **took** treatment | No;  Yes, but not offered hepatitis C treatment;  Yes, offered hepatitis C treatment but didn’t take treatment;  Yes, offered hepatitis C treatment and **took** treatment | No;  Yes, but not offered hepatitis C treatment;  Yes, offered hepatitis C treatment but didn’t take treatment; Yes, offered hepatitis C treatment and waiting to start;  Yes, offered hepatitis C treatment and **took** treatment; |
| Received treatment for HCV | Response to question above | Yes, and been **given** medicine for hepatitis C | Yes, and been **given** medicine for hepatitis C | Yes, and been **given** medicine for hepatitis C | Yes, and been **given** medicine for hepatitis C | Yes, and been **given** medicine for hepatitis C | Yes, and been **given** medicine for hepatitis C | Yes, offered hepatitis C treatment and took treatment | Yes, offered hepatitis C treatment and took treatment | Yes, offered hepatitis C treatment and **took** treatment |

Supplementary Table 2. Numbers and percentages of UAM respondents included in this study, according to operational delivery network (ODNs) and time period.

| Operational delivery network (ODN) | | 2011-2019  N=12320 | | Pre-DAA  N=3949 | | Prioritised DAA  N= 2616 | | Unrestricted DAA  N= 3949 | |
| --- | --- | --- | --- | --- | --- | --- | --- | --- | --- |
|  |  | N | % | N | % | N | % | N | % |
| 1 | North East & Cumbria | 1315 | 10.7 | 681 | 11.8 | 288 | 11.0 | 346 | 8.8 |
| 2 | Greater Manchester & Eastern Cheshire | 433 | 3.5 | 162 | 2.8 | 130 | 5.0 | 141 | 3.6 |
| 3 | Cheshire & Merseyside | 501 | 4.1 | 244 | 4.2 | 146 | 5.6 | 111 | 2.8 |
| 4 | South Yorkshire | 354 | 2.9 | 149 | 2.6 | 77 | 2.9 | 128 | 3.2 |
| 5 | Humberside and North Yorkshire | 230 | 1.9 | 78 | 1.4 | 0 | 0.0 | 152 | 3.9 |
| 6 | West Yorkshire | 895 | 7.3 | 515 | 9.0 | 189 | 7.2 | 191 | 4.8 |
| 7 | Lancashire and South Cumbria | 465 | 3.8 | 197 | 3.4 | 112 | 4.3 | 156 | 4.0 |
| 8 | Leicester | 80 | 0.7 | 0 | 0.0 | 18 | 0.7 | 62 | 1.6 |
| 9 | Birmingham | 1294 | 10.5 | 651 | 11.3 | 211 | 8.1 | 432 | 10.9 |
| 10 | Nottingham | 1028 | 8.3 | 551 | 9.6 | 163 | 6.2 | 314 | 8.0 |
| 11 | Eastern Hepatitis Network | 981 | 8.0 | 374 | 6.5 | 306 | 11.7 | 301 | 7.6 |
| 12 | West London | 306 | 2.5 | 143 | 2.5 | 63 | 2.4 | 100 | 2.5 |
| 13 | North Central London | 338 | 2.7 | 131 | 2.3 | 33 | 1.3 | 174 | 4.4 |
| 14 | Barts | 499 | 4.1 | 328 | 5.7 | 96 | 3.7 | 75 | 1.9 |
| 15 | South Thames Hepatitis Network | 453 | 3.7 | 150 | 2.6 | 85 | 3.3 | 218 | 5.5 |
| 17 | Sussex Hepatology Network | 552 | 4.5 | 323 | 5.6 | 65 | 2.5 | 164 | 4.2 |
| 18 | Oxford University Hospitals NHS Trust | 442 | 3.6 | 242 | 4.2 | 49 | 1.9 | 151 | 3.8 |
| 19 | Wessex Hep C ODN | 734 | 6.0 | 202 | 3.5 | 180 | 6.9 | 352 | 8.9 |
| 20 | Bristol and Severn Hep C ODN | 992 | 8.1 | 403 | 7.0 | 296 | 11.3 | 293 | 7.4 |
| 21 | South West Peninsula Hepatitis C ODN | 254 | 2.1 | 175 | 3.0 | 39 | 1.5 | 40 | 1.0 |
| 22 | Kent Network via Kings | 174 | 1.4 | 56 | 1.0 | 70 | 2.7 | 48 | 1.2 |

Supplementary Table 3. Unadjusted and adjusted odds ratios of recent HCV testing among all participants for 2018-2019.

|  |  | **OR (95%CI)** | **P-Value** | **aOR (95%CI)** | **P-value** |
| --- | --- | --- | --- | --- | --- |
| Ever been homeless | No | Ref |  | Ref |  |
|  | Yes, not in past year | 1.38  (1.09-1.76) | 0.008 | 1.35  (1.03-1.77) | 0.028 |
|  | Yes, in past year | 1.64  (1.38-1.94) | <0.001 | 1.56  (1.26-1.93) | <0.001 |
| Ever been in prison | No | Ref |  | Ref |  |
|  | Yes, not in past year | 1.15  (0.93-1.43) | 0.197 | 0.92  (0.72-1.17) | 0.474 |
|  | Yes, in past year | 1.87  (1.51-2.31) | <0.001 | 1.44  (1.13-1.82) | 0.003 |
| Ever used a needle and syringe programme | Yes, in past year | Ref |  | Ref |  |
|  | Yes, not in past year | 0.72  (0.51-1.03) | 0.076 | 0.72  (0.50-1.02) | 0.061 |
|  | No | 0.53  (0.38-0.73) | <0.001 | 0.66  (0.42-1.03) | 0.065 |
| OAT use | Currently prescribed | Ref |  | Ref |  |
|  | Previously prescribed | 0.78  (0.60-1.01) | 0.063 | 0.678  (0.49-0.91) | 0.010 |
|  | No | 0.54  (0.42-0.69) | <0.001 | 0.55  (0.42-0.73) | <0.001 |
| First injected during preceding three years | More than three years ago | Ref |  | Ref |  |
|  | In last three years | 0.75  (0.58-0.97) | 0.027 | 0.89  (0.67-1.17) | 0.400 |
| Used a GP in past year | | 1.16  (0.94-1.42) | 0.167 | 1.09  (0.85-1.40) | 0.482 |
| Used A&E in past year | | 1.33  (1.14-1.55) | <0.001 | 1.24  (1.01-1.52) | 0.037 |
| Used sexual health service in past year | | 1.61  (1.17-2.21) | 0.003 | 1.57  (1.04-2.37) | 0.031 |
| Used walk-in service in past year | | 1.26  (1.11-1.44) | <0.001 | 1.14  (0.95-1.33) | 0.166 |
| Used pharmacy in past year | | 1.23  (1.00-1.51) | 0.054 | 1.07  (0.86-1.33) | 0.554 |
| Used a dentist in past year | | 1.15  (0.97-1.37) | 0.115 | 1.05  (0.90-1.22) | 0.526 |
| Used prison health service in past year | | 1.58  (1.26-1.99) | <0.001 | 1.25  (0.97-1.61) | 0.079 |

Supplementary Table 4. Unadjusted odds ratios for each step of the cascade of care among ever HCV treatment eligible participants, according to time period.

|  |  | OR (95%CI) | P-value |
| --- | --- | --- | --- |
| Ever tested | Pre-DAAs | 1 |  |
|  | Prioritised DAAs | 0.83 (0.61-1.14) | 0.258 |
|  | Unrestricted DAAs | 0.96 (0.71-1.28) | 0.761 |
| Tested positive | Pre-DAAs | 1 |  |
|  | Prioritised DAAs | 0.93 (0.78-1.12) | 0.453 |
|  | Unrestricted DAAs | 1.12 (0.95-1.32) | 0.178 |
| Seen a specialist nurse or doctor | Pre-DAAs | 1 |  |
|  | Prioritised DAAs | 0.97 (0.81-1.16) | 0.710 |
|  | Unrestricted DAAs | 1.52 (1.29-1.78) | <0.001 |
| Ever treated | Pre-DAAs | 1 |  |
|  | Prioritised DAAs | 1.17 (0.91-1.52) | 0.225 |
|  | Unrestricted DAAs | 2.40 (1.95-2.96) | <0.001 |

Supplementary Figure 1a. HCV cascade of care in the Unrestricted DAAs time period, according to homelessness.

Supplementary Figure 1b. HCV cascade of care in the Unrestricted DAAs time period, according to OAT use.

Supplementary Figure 1c. HCV Cascade of care in the Unrestricted DAAs time period, according to NSP use.

Supplementary Table 5. Unadjusted odds ratios for each step of the HCV cascade of care among ever HCV treatment eligible participants, according to homelessness status, for the Unrestricted DAAs time period.

|  |  | OR (95%CI) | P-value |
| --- | --- | --- | --- |
| Ever tested | Never homeless | 1 |  |
|  | Ever homeless but not in last year | 3.74 (1.54-9.06) | 0.003 |
|  | Homeless in last year | 1.93 (1.27-2.93) | 0.002 |
| Tested positive | Never homeless | 1 |  |
|  | Ever homeless but not in last year | 2.03 (1.43-2.89) | <0.001 |
|  | Homeless in last year | 1.22 (0.89-1.66) | 0.215 |
| Seen a specialist nurse or doctor | Never homeless | 1 |  |
|  | Ever homeless but not in last year | 1.80 (1.14-2.83) | 0.011 |
|  | Homeless in last year | 1.00 (0.71-1.41) | 0.988 |
| Ever treated | Never homeless | 1 |  |
|  | Ever homeless but not in last year | 1.12 (0.79-1.59) | 0.527 |
|  | Homeless in last year | 0.59 (0.44-0.80) | 0.001 |

Supplementary Table 6. Unadjusted odds ratios for each step of the HCV cascade of care among ever HCV treatment eligible participants, according to OAT use, for the Unrestricted DAAs time period.

|  |  | OR (95%CI) | P-value |
| --- | --- | --- | --- |
| Ever tested | Currently prescribed OAT | 1 |  |
|  | Ever prescribed OAT, not current | 1.38 (0.66-2.87) | 0.390 |
|  | Never prescribed OAT | 0.13 (0.08-0.20) | <0.001 |
| Tested positive | Currently prescribed OAT | 1 |  |
|  | Ever prescribed OAT, not current | 0.87 (0.61-1.23) | 0.430 |
|  | Never prescribed OAT | 0.26 (0.15-0.45) | <0.001 |
| Seen a specialist nurse or doctor | Currently prescribed OAT | 1 |  |
|  | Ever prescribed OAT, not current | 0.79 (0.59-1.05) | 0.109 |
|  | Never prescribed OAT | 0.27 (0.17-0.42) | <0.001 |
| Ever treated | Currently prescribed OAT | 1 |  |
|  | Ever prescribed OAT, not current | 0.59 (0.41-0.85) | 0.004 |
|  | Never prescribed OAT | 0.24 (0.11-0.52) | <0.001 |

Supplementary Table 7. Unadjusted odds ratios for each step of the HCV cascade of care among ever HCV treatment eligible participants, according to NSP use, for the Unrestricted DAAs time period.

|  |  | OR (95%CI) | P-value |
| --- | --- | --- | --- |
| Ever tested | Used NSP in last year | 1 |  |
|  | Ever used NSP, not in last year | 1.44 (0.48-4.33) | 0.520 |
|  | Never used NSP | 0.25 (0.07-0.82) | 0.022 |
| Tested positive | Used NSP in last year | 1 |  |
|  | Ever used NSP, not in last year | 1.29 (0.72-2.30) | 0.397 |
|  | Never used NSP | 0.52 (0.20-1.37) | 0.187 |
| Seen a specialist nurse or doctor | Used NSP in last year | 1 |  |
|  | Ever used NSP, not in last year | 1.59 (0.90-2.81) | 0.112 |
|  | Never used NSP | 0.64 (0.27-1.51) | 0.311 |
| Ever treated | Used NSP in last year | 1 |  |
|  | Ever used NSP, not in last year | 1.62 (1.06-2.47) | 0.025 |
|  | Never used NSP | 0.63 (0.26-1.51) | 0.297 |

Supplementary Table 8. Unadjusted and adjusted odds ratios for HCV treatment among ever HCV treatment eligible individuals for 2018-2019.

|  |  | **OR (95%CI)** | **P-Value** | **aOR (95%CI)** | **P-value** |
| --- | --- | --- | --- | --- | --- |
| Age group | 18-29 | Ref |  | Ref |  |
|  | 30-39 | 0.75  (0.42-1.35) | 0.338 | 0.72  (0.40-1.27) | 0.251 |
|  | 40-49 | 0.85  (0.43-1.67) | 0.638 | 0.66  (0.35-1.25) | 0.197 |
|  | 50+ | 1.94  (0.84-4.48) | 0.120 | 1.49  (0.74-2.96) | 0.262 |
| Ever been homeless | No | Ref |  | Ref |  |
|  | Yes, not in past year | 1.14  (0.74-1.75) | 0.546 | 1.09  (0.73-1.62) | 0.671 |
|  | Yes, in past year | 0.59  (0.41-0.86) | 0.006 | 0.60  (0.39-0.93) | 0.021 |
| Ever been in prison | No | Ref |  | Ref |  |
|  | Yes, not in past year | 1.20  (0.84-1.70) | 0.311 | 1.05  (0.73-1.50) | 0.803 |
|  | Yes, in past year | 0.95  (0.61-1.46) | 0.804 | 1.06  (0.67-1.67) | 0.809 |
| Ever used a needle and syringe programme | Yes, in past year | Ref |  | Ref |  |
|  | Yes, not in past year | 1.85  (1.16-2.93) | 0.010 | 1.54  (0.91-2.62) | 0.108 |
|  | No | 0.45  (0.12-1.71) | 0.243 | 0.27  (0.05-1.48) | 0.132 |
| OAT use | Currently prescribed | Ref |  | Ref |  |
|  | Previously prescribed | 0.51  (0.32-0.80) | 0.004 | 0.58  (0.35-0.94) | 0.028 |
|  | No | 0.23  (0.08-0.66) | 0.006 | 0.29  (0.11-0.81) | 0.018 |
| First injected during preceding three years | More than three years ago | Ref |  | Ref |  |
|  | In last three years | 0.23  (0.11-0.50) | <0.001 | 0.31  (0.15-0.64) | 0.001 |
